# Impact of multiple performance feedback and regional institutional development on enterprises’ exploratory innovation

**DOI:** 10.3389/fpsyg.2022.982211

**Published:** 2022-09-30

**Authors:** Xin Su, Wenxiu Fu

**Affiliations:** School of Business and Administration, Shandong University of Finance and Economics, Jinan, Shandong, China

**Keywords:** historical performance feedback, industry performance feedback, regional institutional development, exploratory innovation, corporate behavior

## Abstract

With the increasing uncertainty in the external environment, exploratory innovation has gradually become the key path for enterprises to obtain core competitiveness and achieve sustainable growth. According to the behavioral theory of the firm, performance feedback is an essential driving factor affecting corporate innovation decisions. However, previous studies have ignored the consistency or inconsistency between historical and industry performance feedback, and its impact on exploratory innovation. Based on the data of Chinese companies listed from 2008 to 2019, this paper explores the impact of consistency and inconsistency between historical and industry performance feedback on enterprises’ exploratory innovation. In the cases of consistency, this study finds that the scenario of historical performance shortfall-industry performance shortfall is more likely to promote enterprises’ exploratory innovation than the industry performance surplus-historical performance surplus; in the cases of inconsistency, compared with historical performance surplus-industry performance shortfall, the scenario of historical performance shortfall-industry performance surplus is more likely to promote enterprises’ exploratory innovation. Further research shows that regional institutional development enhances these relationships. This study enriches the driving factors of enterprises’ exploratory innovation from the perspective of multiple performance feedback, which can provide decision-making references for enterprises’ exploratory innovation strategies.

## Introduction

In the context of increasing uncertainty, volatility, complexity, and ambiguity in the external environment, technological innovation has become the core driving force for enterprises to enhance competitiveness and achieve sustainable development (Bonaime et al., 2018; Su et al., 2020; Honig and Samuelsson, 2021). Exploratory innovation (EI) is essential for enterprises to adapt to dynamic changes in the external environment and maintain a continuous competitive advantage (Tian and Wang, 2014; Tian et al., 2020; Wang N. et al., 2021). EI disrupts organizational inertia by introducing new knowledge, methods, and designs, making it conducive to the fundamental transformation of production technology (Slavova and Jong, 2021). EI can not only change the original performance and characteristics of products and create new products for enterprises, but can also open up new consumer markets and help enterprises obtain excess profits (Gao et al., 2015; Lin and Patel, 2019). Especially in the shortening product life cycle and rapidly changing market competition patterns, EI is conducive to meeting the enterprises’ development needs and promoting enterprises’ sustainable and healthy growth (Wang et al., 2020).

According to the behavioral theory of the firm, an enterprise is an organizational system oriented by a goal decision that will establish a reference point (aspiration level) that satisfies the decision-maker when evaluating the operating status of the enterprise (Cyert and March, 1963; Chen et al., 2022) and adjust the organizational decision according to the gap between the actual performance and the aspiration level to change the enterprise innovation strategy (Zajac and Kraatz, 1993). Some scholars consider that enterprises usually become problem-oriented, and actively seek solutions to promote innovation and change the current situation of poor management when the actual performance is lower than the aspiration level (Gaba and Joseph, 2013; Choi et al., 2019). Conversely, when the actual performance is higher than the aspiration level, enterprises usually continue with the previous strategy rather than risk innovation (Barnett and Pontikes, 2008).

Other scholars hold the opposite view that when the actual performance exceeds the aspiration level, abundant redundant resources provide sufficient funds for innovation activities, trigger the redundancy-driven search mechanism of enterprises, and improve the enthusiasm of enterprises for innovation (Parker et al., 2017; Eggers and Suh, 2019). However, enterprises face greater resource constraints when the actual performance is lower than the aspiration level and take conservative or prudent measures to inhibit innovation (Chng et al., 2015). There is no consensus in the literature regarding the impact mechanism of performance feedback on enterprise innovation. Most studies regard enterprise innovation as a comprehensive concept and ignore the differences in innovation behaviors with different characteristics. Enterprise innovation depends on the profit return effect of innovation behavior (Wang and Wang, 2020). Enterprises’ EI is characteristic of high-risk and high-profit, which bring new technologies to enterprises, seize the market, and obtain excess returns. Enterprises’ EI has a more profound impact on the long-term sustainable development of enterprises (Wang et al., 2019; Yi et al., 2022). Considering the high-risk and high-profit characteristics of EI, enterprises may follow the decision-making logic of “performance shortfall leads to change” and actively carry out EI, as the actual performance is lower than the aspiration level, to change the performance dilemma. Enterprises may follow the decision-making logic of “performance surplus leads to strategy persistence” to avoid the high opportunity cost of innovation decision transformation, as the actual performance is higher than the aspiration level. Thus, we explore the impact of performance feedback on enterprises’ EI and provide theoretical references for enterprise innovation strategies.

In the actual decision-making process of enterprises, decision-makers judge the operating status of enterprises based on both historical and industry aspiration levels rather than a single aspiration level (Kim et al., 2015). There are essential differences in the information contained in historical and industry aspiration levels. The historical aspiration level represents the enterprise’s operation and management goal, while the industry aspiration level represents the enterprise’s competitive position within the market. However, analyzing the relationship between performance feedback and enterprise innovation by focusing on a single reference point in a historical or industry context is too idealistic to describe the connotation of performance feedback and may weaken the explanatory power of the performance feedback mechanism (Wang and Lou, 2020; Chung and Shin, 2021). We can obtain a more complete insight into the relationship between performance feedback and enterprises’ EI only by distinguishing between historical and industry performance feedback and examining how their complex interaction influences EI (Lv et al., 2019).

Complex performance feedback situations are the norm in corporate decision-making, which run through the entire process. According to the multiple performance expectations of historical and industry contexts, there are two possibilities regarding consistency and inconsistency, historical performance shortfall-industry performance shortfall and historical performance surplus-industry performance surplus; historical performance shortfall-industry performance surplus and historical performance surplus-industry performance shortfall. Especially in the cases of inconsistency, the business status of the enterprise cannot be defined as loss or gain, and decision-makers need to analyze the above information (Joseph and Gaba, 2015). Inconsistent performance feedback is ambiguous, and decision-makers must clearly analyze the state. Decision-makers make different judgments according to inconsistent feedback information affecting the enterprises’ EI.

We systematically explore the impact of the consistency and inconsistency between historical and industry performance feedback on EI based on the behavior theory of the firm. We believe that, in the cases of consistency, the historical performance shortfall-industry performance shortfall is more likely to promote enterprises’ EI than industry performance surplus-historical performance surplus. When both the historical and the industry performance feedback are in shortfall, decision-makers recognize that there are some problems within the enterprise. This creates an impetus within enterprises to change the status quo and enhance competitive advantage, and necessitates enterprises to implement high-profit EI (Lu and Wong, 2019). However, enterprises have stable revenue expectations when both the historical and the industry performance feedback are surplus. In this context, although enterprises have ample redundant funds, the opportunity cost of strategic adjustment is high to change the existing innovation decisions, disrupt organizational practices, and carry out EI with high-risk and high-profit. Therefore, decision-makers continue to implement the past innovation strategies, which is not conducive to EI.

In the cases of inconsistency, historical performance shortfall-industry performance surplus is more likely to promote EI than historical performance surplus-industry performance shortfall. Compared with industry performance feedback, historical performance feedback reflects the management ability of enterprise decision-makers more directly. In the cases of historical performance shortfall-industry performance surplus, decision-makers consider that historical performance shortfall may damage their reputation. To prove their leadership ability, enterprise managers actively use the advantages brought by the industry performance surplus to solve existing problems. Consequently, decision-makers actively seek organizational innovation, enhance enterprises’ EI, and obtain more benefits, thereby reducing the historical performance shortfall and maintaining their reputation and image. In the cases of historical performance surplus-industry performance shortfall, decision-makers attribute the industry performance shortfall to the uncertainty factors in the external environment, avoiding personal image and evaluation damage, which stimulates self-enhancement motivation (Jordan and Audia, 2012; Audia et al., 2015). Decision-makers interpret the historical performance surplus-industry performance shortfall as a state of benefit, strengthening the strategic rigidity, which is not conducive to the enterprises’ EI.

Furthermore, the impact of consistency and inconsistency of performance feedback on EI may be restricted by the external environment, primarily regional institutional development. Regional institutional environment development change decision-makers’ understanding and response to feedback signals and affect the relationship between performance feedback and enterprises’ EI (Ben-Oz and Greve, 2015; Su and Si, 2015). As an important form of the soft power of national or regional economic development, regional institutional development (RI) provide ideal conditions for enterprises’ technological innovation by optimizing the policy, market, and factor environments, thereby changing the mechanism of performance feedback on enterprises’ EI (Davis and North, 1970). On the one hand, RI can reduce the organizational transaction cost of the enterprise’s problem searching activities and effectively alleviate the level of information asymmetry between enterprises, improving the efficiency of enterprise resource allocation, promoting the flow and integration of enterprise innovation elements, and thus promote EI (Wu et al., 2019). On the other hand, RI provide a fair, competitive market, thereby enhancing the confidence of decision-makers in innovation in the case of performance feedback shortfall, and stimulating the vitality of enterprises’ EI (Yang et al., 2012). In conclusion, we believe that the impact of performance feedback consistency and inconsistency on enterprises’ EI is enhanced with the improvement of the RI level.

China is the world’s second-largest economy, and innovation is the core of China’s modernization drive. To build an innovation-oriented country, it is necessary to realize high-quality development of the national economy so that enterprises conduct EI and improve their core competitiveness actively. In addition, China is in a critical period of transformation and upgrading, and the development of the market mechanism is still incomplete. As the main component of the modern market economy, enterprises are the key force for the country to improve its innovation capability and implement the innovation-driven development strategy. Thus, China provides a suitable environment for examining the EI of enterprises.

Based on the sample data of Chinese companies listed from 2008 to 2019, we examine the impact of multiple performance feedback on enterprises’ EI. This study finds that after controlling for variables at the level of corporate characteristics and corporate governance, the empirical evidence for the abovementioned theoretical viewpoints holds. In the cases of consistency of performance feedback, historical performance shortfall-industry performance shortfall is more likely to promote enterprises’ EI. In the cases of inconsistency, historical performance shortfall-industry performance surplus is more likely to promote enterprises’ EI. RI strengthens the abovementioned positive relationship. In addition, we replace the measurement indicator of the independent variable, change the measurement method of the independent variable, and used the systematic GMM model to perform an endogeneity test, and the results remain robust.

In summary, this paper has three theoretical contributions: First, we enrich relevant research on the driving factors of enterprises’ EI from the perspective of multiple performance feedback. Considering the impact of performance feedback on enterprise innovation prior studies mainly focused on a single reference point, namely, historical performance feedback or industry performance feedback (Chen et al., 2021); few studies have explored the impact of consistency between history and industry performance feedback on enterprise innovation (Lucas et al., 2015; Lv et al., 2019). In the cases of inconsistency, negative historical and positive industry performance feedback positively affected the enterprises’ innovation (Ye and Zhao, 2021). Enterprises’ EI can bring new technologies and products and has a high-value return effect. Existing studies lack an in-depth discussion on the relationship between multiple performance feedback and enterprises’ EI. To fill this gap, we include both historical and industry performance feedback in the same research framework to further analyze the consistency and inconsistency of situation combinations of multiple performance feedback influences on EI decision-making.

Second, our research expands the existing theoretical model of performance feedback and enterprises’ EI decision-making. Previous studies focused on the influence of internal factors on the relationship between performance feedback and enterprise innovation while ignoring the vital role of the external environment for enterprises’ survival and development (Zhong et al., 2022). We explore the contingency effect of RI on the impact of performance feedback on EI, which could help enterprise decision-makers pay more attention to changes in external RI and optimize enterprises’ EI strategy.

Third, this study provides concrete empirical evidence of enterprises’ EI decision-making. From the multiple reference points of historical and industry performance expectation, we explore the differential influences of combination situations with multiple performance feedback on EI of enterprises, providing a practical reference for enterprises to improve EI strategy and achieve sustainable development.

The rest of this paper is organized as follows. In the section “Theoretical Analysis and Hypotheses,” we discuss the hypothesis development. In the section “Research Design,” we introduce the data and methods. In the section “Results,” we discuss the empirical results, and in the last section “Conclusion and Discussion,” we conclude the study.

## Theoretical analysis and hypotheses

### Multiple performance feedback

Bounded rational decision-makers usually judge the current operating state of an organization based on experience to simplify decision-making. In evaluating the actual performance, a satisfactory reference point, namely the aspiration level, is determined and enterprise decisions are adjusted according to the reference point (Hart and Moore, 2008). Reference points for decision-making are mainly affected by two factors: their historical performance and the average performance of other organizations in the same industry (Kim et al., 2015). Decision-makers of enterprises explore corresponding innovative decision-making schemes and adjust rules based on their historical performance feedback and industry performance feedback (Denrell and March, 2001). An enterprise’s actual performance that is higher than the aspiration level is called performance feedback surplus, while its performance lower than the aspiration level is called performance feedback shortfall. Decision-makers with bounded rationality define performance feedback surplus as the benefits state of the organization, and performance feedback shortfall as the loss state.

When an enterprise has a multiple reference point for historical performance expectation and industry performance expectation, there are two possibilities (consistency and inconsistency) in the performance evaluation results, presenting four different combinations of scenarios, as shown in Table 1. The consistency of performance feedback means that the two groups of performance feedback signals are in the same direction, such as in the scenario of historical performance surplus-industry performance surplus and the scenario of historical performance shortfall-industry performance shortfall. When the feedback of historical and industry performance is presented in the scenarios of consistency, the dual information feedback standard provides a clear, accurate and credible signal for enterprises, and decision-makers of enterprises do not need to further interpret the feedback results but need to respond quickly. According to consistent performance feedback signals, decision-makers timely adjust the current strategic decision, prompt enterprises to quickly search and reallocate limited resources, and seize opportunities to establish advantages, which is conducive to the development of EI activities of enterprises (Lucas et al., 2015). Inconsistent performance feedback implies that the two groups of performance feedback signals are in opposite directions, such as in the scenario of historical performance surplus-industry performance shortfall and the scenario of historical performance shortfall-industry performance surplus. When the feedback of historical and industry performance is presented inconsistently, it is difficult for enterprises to clearly define the current business situation, which increases the difficulty of decision-making and reduces the adaptive change response of enterprises (Joseph and Gaba, 2015). In the face of consistent and inconsistent performance feedback signals, there is a significant difference in the decision-making behavior of enterprises. It is necessary to further discuss the relationship between performance feedback and EI of enterprises under the two sets of performance reference points of history and industry. Therefore, we explore the differential impact of combinations of multiple performance feedback consistency and inconsistency on enterprises’ EI.

**Table 1:** The cases of historical-industry performance feedback.

Concept	Cases	
Consistency	Historical performance surplus-industry performance surplus	Historical performance shortfall-industry performance shortfall
Inconsistency	Historical performance surplus-industry performance shortfall	Historical performance shortfall-industry performance surplus

### Multiple performance feedback and enterprises’ exploratory innovation

In the cases of consistency between historical and industry performance feedback, multiple performance reference points provide enterprises with more accurate feedback signals. When both the historical and the industry performance feedback are in surplus, the enterprise is defined as being in an absolute benefit state. This situation leads to behavioral inertia in enterprises, which is not conducive to EI. First, the performance feedback surplus proves to some extent that the current strategic decision is suitable for the development of enterprises and bring higher profits for enterprises. At the same time, considering stakeholders’ demands for organizational stability, enterprises tend to give up EI activities with high risks and high returns to maintain existing benefits (Lin, 2014). EI needs great reform, requiring organizational breakthrough and transformation. In particular, the opportunity cost of EI is higher when the enterprise has stable income expectations. Even in the cases of historical performance surplus-industry performance surplus, enterprises have abundant redundant resources. Consequently, they are unwilling to change the existing innovation strategy to carry out EI. Second, performance feedback surplus enhances decision-makers’ perception of business benefits and breeds overconfidence, thus strengthening the soundness of experience and organizational practices. Decision-makers of enterprises in this situation assume that past decisions and experience are conducive to enterprise growth, and become unwilling to increase R&D investment and change the existing innovation strategy planning. To avoid unnecessary losses caused by risky changes or innovative investments, enterprises may choose initial strategic planning to maintain the status quo (Joseph et al., 2016; Yang et al., 2017). Third, when the business performance is higher than the aspiration level, the enterprise lacks the motivation to search for external information, which reduces the scope of the organizational search and is not conducive to generating valuable new knowledge and new ideas. This is mainly because the previous successful experience reduces the perception of decision-makers of environmental uncertainty risks. Decision-makers believe that the current operation and management decisions are in line with the development of enterprises, neglecting the in-depth mining of external information, and reducing enterprises’ ability for EI.

When both the historical and the industry performance feedback are in shortfall, it indicates that the actual operating performance of the enterprise is lower than the aspiration level and that the organization’s current business strategy, resource allocation, or market competition mechanism is not perfect; further, the enterprise is defined as being in an absolute loss state. Multiple performance feedback shortfall makes decision-makers realize that there are problems in organizational operation. Thus, enterprises improve their core competitiveness and obtain higher returns only by disrupting organizational inertia and choosing EI with high risks and high returns (Saraf et al., 2021).

On the one hand, a performance lower than the aspiration level will trigger the enterprise problematic search mechanism, according to the existing problems targeted innovation strategy adjustment. Decision-makers optimize the internal resource allocation of enterprises, acquire external novel knowledge, and stimulate a forward-looking thinking mode, thereby enhancing enterprises’ EI to adapt to customer and product market competition and improving enterprise benefit ability (Greve, 2003). Enterprises obtain information, knowledge, and technology in various ways, and actively integrate and innovate knowledge, adjust the operation mode of enterprises promptly, and improve the ability of enterprises to deploy and utilize resources, which is conducive to optimizing the existing production and operation processes and improving the efficiency of production technology, putting more operating profits into innovation and research and development, and strengthening enterprises’ EI capabilities.

On the other hand, when the performance feedback shortfall threatens the organization’s reputation and external legitimacy, decision-makers of enterprises need to make reasonable explanations. When the performance of an organization continues to decline and decision-makers fail to propose corresponding solutions, stakeholders will question the organizational development strategy (Desai, 2014). The simultaneous existence of internal and external crisis threats forces enterprise decision-makers to rethink the organization’s business strategy and innovation decisions and actively promote EI of enterprises to seize the opportunity to occupy the market (Oliver, 1992; O’Brien and David, 2014; Xue et al., 2021). In addition, to decrease their performance shortfall, decision-makers of enterprises tend to prefer high-profit and high-risk projects and try to change the status quo of adventure and innovation investment in a willingness to strengthen which is advantageous to the enterprise to reposition in the market. They further try to increase R&D investment, develop new products, obtain higher usefulness or value of innovation, and strengthen enterprises’ EI. Therefore, we propose the following hypothesis H1:

*H1*: In the cases of consistency of performance feedback, compared with the historical performance surplus-industry performance surplus, historical performance shortfall-industry performance shortfall is more likely to promote enterprises’ EI.

Inconsistent performance feedback includes two scenarios: historical performance surplus-industry performance shortfall and historical performance shortfall-industry performance surplus. The inconsistent feedback information can help them identify the operating state and industry situation under the multiple performance evaluation criteria to conduct targeted innovation strategy adjustments (Zhang and Gong, 2018). Compared with industry performance feedback, historical performance feedback reflects the decision-maker’s management ability more directly. The historical performance feedback is in surplus, while the industry performance feedback is in shortfall, indicating that although the enterprise does not meet the average performance of the industry, its performance is higher than its performance expectation. Decision-makers believe that the enterprise is in a rising and progressive trend, defined as a state of relative benefit. In this scenario, decision-makers tend to prefer to pursue the favorable direction, hide the negative evaluation of industry performance shortfall, and stimulate the motivation of self-enhancement. Decision-makers are driven by the psychological need to affirm themselves and avoid negative assessments to maintain their reputation and image. Decision-makers regard the feedback status of historical performance surplus of enterprises and industry performance shortfall in a positive way, which leads to the strategic rigidity of enterprises and is not conducive for the breakthrough innovation of enterprises (Audia et al., 2015; Wan et al., 2022).

In addition, the scenario of the historical performance feedback being in a surplus indicates that the current enterprise innovation strategy will lead to higher profits in the future. Enterprises will continue to keep the original technology, skills, and management style, and discontinue developing new products and technology, and expand product lines to meet more customer needs, thereby lowering the EI of enterprises.

When the historical performance shortfall and industry performance surplus occur simultaneously, it indicates that the actual performance is higher than the industry average but does not achieve the self-expected goals, and the enterprise is said to be in a relative loss state. Decision-makers may actively perform EI to obtain excess returns in order to reduce the current situation of historical performance shortfall and maintain their reputation and image proving their leadership and management ability (Kacperczyk et al., 2015). On the one hand, the historical performance shortfall leads to inadequate resource investment, and the original competitive advantage is difficult to sustain. Under the influence of the reputation incentive mechanism, enterprise decision-makers will actively change the existing business strategy and seek new competitive advantages to narrow the historical performance feedback shortfall and stabilize the position of enterprises in the industry (Yu et al., 2022). Here, the enterprise actively implements a series of breakthrough changes and perform technology, product, and service innovations to enhance the EI of the enterprise (Xiao et al., 2021). Decision-makers conduct problematic search activities within the organization, actively update and reorganize existing knowledge, skills, and experience, reduce production and operating costs, and improve product skills, which are conducive to disrupting the conventional cognitive model of the organization and propose improvements. From a new knowledge perspective, they offer solutions to improve the status quo of poor performance, thereby enhancing the level of EI of enterprises.

On the other hand, the historical performance shortfall-industry performance surplus may also indicate that with the increasing uncertainty of the external environment, the development of the whole industry is depressed. Although the industry performance is in a surplus, the enterprise cannot be said to be in an excellent state. The decline or recession of the overall industry can cause enterprise decision-makers to be aware of crises, improve enterprises’ risk tolerance, enhance the confidence and ability of enterprises to seize the market, and stimulate enterprises to perform EI (Blagoeva et al., 2020). Therefore, we propose the following hypothesis H2:

*H2*: In the cases of inconsistency of performance feedback, the scenario of historical performance shortfall-industry performance surplus is more likely to promote EI than the scenario of historical performance surplus-industry performance shortfall.

### The moderating effect of regional institutional development

New institutional economics indicates that one of the critical factors affecting technological progress and innovation behavior is the development of the regional institutional environment. The development of a regional system includes a series of external environmental factors that include policy, market, and factor environment that enterprises face in the production and management domains. In particular, China is in the process of economic transformation and upgrading, and the interaction between changes in the regional institutional environment and organizational strategic decision-making is more closely related. RI is an essential factor affecting corporate innovation decision-marking (Alam et al., 2019).

RI can not only ease the impact of uncertain factors on enterprises and give full play to the external governance effect, stimulating enterprises to perform EI activities, but can also reduce their operating cost by optimizing the allocation of innovation resources, thus increasing innovation and helping the sustainable growth of the enterprise (Szczygielski et al., 2017). Specifically, on the one hand, RI provides market signals for enterprises. An excellent institutional development environment means a fair and reasonable market competition atmosphere, enables enterprise decision-makers to obtain more sufficient information, optimize resource allocation, and weaken the impact of information asymmetry. On the other hand, the development of regional institutions allows the positioning of enterprises in the innovation network and reduces the investment risk of EI (Liu et al., 2022). Relevant policies such as direct financial subsidies and low-interest loans effectively reduce the cost of enterprises’ EI and enable them to invest more capital in R&D innovation (Donbesuur et al., 2020).

RI affects decision-makers’ cognition and judgment of performance feedback information. It determines whether an enterprise will perform the EI behavior of active search and disruptive change. In regions with developed institutions, enterprises attach more importance to the guiding role of the performance feedback mechanism in operation decision-making, and can timely adjust strategic decisions based on feedback signals. Especially in the scenario of historical performance shortfall-industry performance shortfall, RI provides more convenience for enterprises’ EI, strengthens the motivation of enterprises’ EI, and enhances the positive relationship between historical performance shortfall-industry performance shortfall and enterprises’ EI (Wang et al., 2015).

On the one hand, when the enterprise is in shortfall, enterprise managers actively disrupt the initial organizational inertia and prevalent management thinking and search for existing problems in the enterprise. RI can effectively reduce the cost of problem-driven search to help enterprises improve the current situation. RI can not only provide enterprises with more external resources and information and reduce the difficulty of obtaining external information, but can also optimize the allocation of resources and improve the efficiency of enterprises in creating new knowledge and technology (Shu et al., 2016). On the other hand, RI brings more institutional environment support to enterprises and increases the risk preference of enterprise decision-makers under the condition of the shortfall. In an optimized institutional environment, the market occupies a dominant position in resource allocation, and relevant policies regulate and constrain the behavior of enterprises, creating a relatively fair and free competitive environment, which is conducive to stimulating the innovation vitality of enterprises. RI provide effective information for the market system of regional development guidance, while perfect factors and the market environment alleviate the enterprise investment risk, promote the flow of innovation between enterprises, and enhance the high-risk, high-income EI ability (Aghion et al., 2005), which helps the enterprise obtain excess returns and long-term competitive advantage. Therefore, the enterprise maintains its reputation and external legitimacy. We propose the following hypothesis H3:

*H3*: In the cases of consistency of performance feedback, RI positively moderates the relationship between the scenario of historical performance shortfall-industry performance shortfall and enterprises’ EI.

When faced with inconsistent performance feedback, especially in the scenario of historical performance shortfall-industry performance surplus, RI can improve the effectiveness of the performance feedback mechanism, strengthen the reputation incentive effect of decision-makers, and enhance the EI of enterprises. In regions where regional institutions are well developed, enterprises face fewer difficulties or obstacles and operate more smoothly, which is conducive to maintaining the reputation of enterprises’ decision-makers and improving their leadership (Tang et al., 2019). When an enterprise is in a state of historical performance shortfall, decision-makers’ reputation is damaged. It is necessary to detect the problems of the enterprise in time and change the state of historical performance shortfall. Thus, the external resources brought by RI reduce the uncertainty of an enterprise’s operations and strengthen the management confidence of decision-makers. The positive environment created by RI for enterprises enhance decision-makers’ enthusiasm to seek innovative breakthroughs and promote enterprises’ EI (Xu et al., 2012).

Additionally, RI reduces decision-makers’ worries about the uncertain industrial environment and improves their risk tolerance, promoting enterprises to actively reform and innovate (Wang Y. et al., 2021). Some enterprise decision-makers believe that the surplus of industry performance does not mean that the enterprise develops well because the uncertainty of the external environment makes the industry competition fluctuate significantly, and causes the whole industry to decline or recession, thereby reducing the credibility of the industry performance feedback. Although enterprises are in the industry performance surplus state, they still need to perform innovation in regions with better RI actively. The development of regional systems is conducive to creating orderly market rules, injecting new vitality into the market, and bringing more development opportunities to enterprises. Market and competition mechanisms become fair, thereby relieving the operating pressure of decision-makers facing environmental uncertainty and enhancing confidence of enterprise development (He et al., 2021; Tian et al., 2022). Under the combined action of positively promoted RI and reverse-promoted performance feedback, enterprises actively seize external opportunities, create new products, and obtain new markets to improve the level of EI of enterprises (Ciftci and Cready, 2011). Therefore, we propose the following hypothesis H4:

*H4*: In cases of inconsistency of perform feedback, RI positively moderates the relationship between the scenario of historical performance shortfall-industry performance surplus and enterprises’ EI.

## Research design

### Sample and data

We selected Chinese A-share listed companies from 2008 to 2019 as the research samples to explore the impact of performance feedback on enterprises’ EI. We chose 2008 as the starting year because China implemented new accounting standards in 2007. To ensure the rationality of sample selection and follow the results of previous studies, we strictly screened research samples according to the following exclusion criteria Zhong et al. (2021): (1) the samples of regulated financial companies such as banks, security companies and insurance companies were excluded leading to the deletion of data of 235 companies; (2) special treatment and particular transfer company samples were excluded leading to the deletion of data of 221 companies; (3) the company samples with serious missing data were removed, leading to 786 companies being excluded. Through the above screening steps, unbalanced panel data of 14,825 listed companies were finally obtained during the sample period, involving 2,313 companies. The basic characteristic data of the enterprise and the characteristic data of the corporate governance level used in this study were obtained from the CSMAR and WIND databases, which are authoritative and comprehensive data sets of Chinese listed firms and widely used by strategic management scholars. In addition, to overcome the influence of outliers and ensure the quality and accuracy of data, a 1% tail reduction was applied to all continuous variables.

### Definition of variables

#### Exploratory innovation

There are many ways to measure EI. Arzubiaga et al. (2019) and Berraies (2019) used the questionnaire survey method to measure the EI of enterprises with relevant items. We referred to the research Gao et al. (2021) and Guan and Liu (2016) to measure EI using the International Patent Classification (IPC) number. Using the IPC number to measure EI can eliminate the subjectivity brought by the questionnaire to a certain extent. EI is an innovative activity that brings new knowledge, technology, and products to the enterprise, and has vital creativity and innovation. The IPC number four represents patent technology of classification, if the patent applied by the enterprise in the current year is different from the IPC number in the previous 5 years. In that case, the number of patents whose classification number is not repeated is recorded as EI (Gilsing et al., 2008). The measurement method is the log of the number of patent applications plus 1.

#### Historical performance feedback and industry performance feedback

Drawing on the practice of Joseph and Gaba (2015), we measured the historical performance feedback (HAP) and industry performance feedback (IAP) by the difference between actual performance and aspiration level. We used exponential smoothing to calculate the historical performance aspiration level (HA) and industry performance aspiration level (IA). The actual performance of enterprise I in period T is $P_{i,t}$ , which is measured by return on assets (ROA). The historical performance expectation of enterprise I in period T is $HA_{i,t}$ . The historical performance desire level can be formulated as $HA_{i,t}$ =a $P_{i,t-1}$ + (1–a) $HA_{i,t-1}$ . The historical performance feedback can be expressed as HAP= $P_{i,t}$ − $HA_{i,t}$ . Based on the same method, $I_{i,t}$ was set the actual median performance of all enterprises in the industry of company I in period T. The industry performance expectation of enterprise I in period T is $IA_{i,t}$ . The IA can be formulated as $IA_{i,t}$ =a $I_{i,t-1}$ +(1−a) $IA_{i,t-1}$ . The industry performance feedback expression is IAP= $P_{i,t}$ − $IA_{i,t}$ ; where *a* is an adjustment parameter between [0,1] and represents the weight between the performance of the current period and the aspiration level of the previous period in the HA level of the current period. This is based on research of Chen (2008), where the weight is designated 0.6 (Zhong et al., 2022).

According to Lucas et al. (2015), we multiplied the feedback values of the two performance dimensions to represent the interaction of different performance feedback. In the cases of consistency, the multiple performance feedback of historical performance shortfall-industry performance shortfall and historical performance surplus-industry performance surplus was defined as a dummy variable HAPIAP1, historical performance shortfall-industry performance shortfall with the expression ( $P_{i,t}$ − $HA_{i,t}$ < 0) × ( $P_{i,t}$ − $IA_{i,t}$ < 0) was defined as 1, and historical performance surplus-industry performance surplus with the expression ( $P_{i,t}$ − $HA_{i,t}$ > 0) × ( $P_{i,t}$ − $IA_{i,t}$ > 0) was defined as 0. In the cases of inconsistency, the multiple performance feedback of historical performance shortfall-industry performance surplus and historical performance surplus-industry performance shortfall was defined as a dummy variable HAPIAP2, historical performance shortfall-industry performance surplus with the expression ( $P_{i,t}$ − $HA_{i,t}$ < 0) × ( $P_{i,t}$ − $IA_{i,t}$ > 0) was defined as 1, historical performance surplus-industry performance shortfall with the expression ( $P_{i,t}$ − $HA_{i,t}$ > 0) × ( $P_{i,t}$ − $IA_{i,t}$ < 0) was defined as 0.

#### Regional institutional development

Regional institutional development (RI) as a comprehensive external environment may play a moderating role in the impact of performance feedback on enterprises’ EI. Gao et al. (2015) used questionnaires to measure the RI environment and divided the institutional environment into formal and informal institutional environments. The formal institutional environment mainly includes innovation policies, procurement policies, tax policies, and the legal environment provided by the government that are conducive to the development of enterprises. The informal institutional environment mainly includes the social and cultural backgrounds. Regional system development mainly includes the policy, market, legal, factor environments, and others. The indexes found in The Report of Market Index by Provinces in China, such as the relationship between the government and the market and the development degree of the product and factor markets and the legal environment, can better reflect the overall level of RI, and can be used as proxy indexes to evaluate RI. Considering the consistency and continuity of the research sample period, we adopted the corresponding indicators in the China Provincial Marketization Index Report published in 2021 by Wang Xiaolu et al. to measure the development level of regional institutions.

#### Control variables

To account for alternative explanations, we include a comprehensive set of control variables from the level of corporate characteristics and governance (Wang et al., 2019; Chu et al., 2021). At the level of company characteristics, firm size denotes the logarithm of the total assets of an enterprise. As an aggregate of different resources, the enterprise scale will directly affect the EI of enterprises. The ownership structure has a significant impact on EI. If the enterprise is a state-owned enterprise, we defined it as 1, and if the enterprise is a non-state-owned enterprise, as 0. The leverage ratio, that is, the ratio of total liabilities to total assets at the end of the period, reflects the debt and risk-bearing capacity of enterprises. Corporate debt brings not only resources to enterprises, also high risks. However, it is not conducive to EI with high-risk characteristics. The fixed asset ratio is the ratio of fixed assets to total assets at the end of the period. The intangible assets ratio refers to the ratio of intangible assets to total assets at the end of the period. Fixed asset ratio and intangible asset ratio can indicate the utilization of enterprise capital, and then affect the breakthrough innovation of enterprises. Precipitate slack resources denote the sum of sales and administrative expenses and the ratio of enterprise operating income. Non-precipitate slack resources are measured by the ratio of current assets to current liabilities. Redundant resources provide enterprises with continuous capital investment to promote enterprise innovation, but different redundant resources may have a different impact on enterprises’ EI.

At the level of corporate governance, board size represents the natural logarithm of the number of directors after adding 1, and the board of directors decides the strategies and their scale influences the EI of enterprises. The ownership concentration refers to the shareholding ratio of the largest shareholder of an enterprise, which reflects the internal control problems of the enterprise. Additionally, we controlled for the individual effect (ID) and time effect (YEAR) to eliminate the factors that do not change with time at the enterprise level and the influence of common time trends on the EI of enterprises.

### Model setting

We constructed the following regression model to test the impact of multiple performance feedback on enterprises’ EI (Liu et al., 2021; Shi et al., 2022) and the moderating role of RI (Xu et al., 2022; Xue et al., 2022; Zhang et al., 2022).


(1) $$EI_{i,t}=a_{0}+a_{1}\mathrm{HAPIAP}1_{i,t}+\mathrm{Contro}l_{i,t}+e_{i,t}$$

(2) $$EI_{i,t}=a_{0}+a_{2}\mathrm{HAPIAP}2_{i,t}+\mathrm{Contro}l_{i,t}+e_{i,t}$$

(3) $$\begin{array}{c} EI_{i,t}=b_{0}+b_{11}\mathrm{HAPIAP}1_{i,t}+b_{12}RI_{i,t}+b_{13}RI_{i,t}\times \\ \mathrm{HAPIAP}1_{i,t}+\mathrm{Contro}l_{i,t}+e_{i,t} \end{array}$$

(4) $$\begin{array}{c} EI_{i,t}=b_{0}+b_{21}\mathrm{HAPIAP}2_{i,t}+b_{22}RI_{i,t}+b_{23}RI_{i,t}\times \\ \mathrm{HAPIAP}2_{i,t}+\mathrm{Contro}l_{i,t}+e_{i,t} \end{array}$$

where $EI_{i,t}$ represents the EI of enterprises with dependent variable, $\mathrm{Contro}l_{i,t}$ represents the control variables, and $e_{i,t}$ is the random error term. $\mathrm{HAPIAP}1_{i,t}$ represents the multiple performance feedback in the cases of consistency, $\mathrm{HAPIAP}2_{i,t}$ represents the multiple performance feedback in the cases of inconsistency, $RI_{i,t}$ represents RI. $RI_{i,t}\times \mathrm{HAPIAP}1_{i,t}$ represents the interaction term between the multiple performance feedback in the cases of consistency and RI, and $RI_{i,t}\times \mathrm{HAPIAP}2_{i,t}$ represents the interaction term between the multiple performance feedback in the cases of inconsistency and RI. In addition, we used a panel fixed-effects model for regression analysis to reduce bias for potential omitted variables.

## Results

Table 2 lists the descriptive statistics of the main variables. The mean value of EI is 2.3428 and the standard deviation is 1.5268, indicating that the average level of EI of enterprise is relatively weak, and there are great differences in the level of EI among the various enterprises; the mean value of historical performance feedback is −0.0065, the standard deviation is 0.0486, the minimum value is −0.4235, and the maximum value is 0.2445; the mean value of industry performance feedback is −0.0002, the standard deviation is 0.0638, the minimum value is −0.4961, and the maximum value is 0.1986, and there are significant differences among different companies. The average value of RI is 8.3730, the maximum value is 12.1900, and the minimum value is 3.1500. The overall level of RI in China is not high, and the RI index of different regions has a big gap. We conducted a variance inflation factor test for all variables in the model, and the VIF value of the model was less than 2. There was no severe multicollinearity interference, indicating that our variable setting was reasonable.

**Table 2:** Descriptive statistics and correlation coefficients.

Variables	Mean	SD	Min	P50	Max	1	2	3	4	5	6	7	8	9	10	11	12	13
1. EI	2.3428	1.5268	0	2.3025	6.8997	1												
2. HAP	−0.0065	0.0486	−0.4235	−0.0007	0.2445	−0.0249^***^	1											
3. IAP	−0.0002	0.0638	−0.4961	0.0007	0.1986	0.0331^***^	0.0674^***^	1										
4. RI	8.3730	2.0849	3.1500	8.3800	12.1900	0.1978^***^	−0.0284^***^	0.0331^***^	1									
5. SCALE	21.9816	1.2592	19.4454	21.7842	26.3684	0.2327^***^	0.0117	0.0148^**^	−0.0036	1								
6. STATE	0.3436	0.4749	0	0	1	−0.0185^***^	0.0370^***^	−0.0884^***^	−0.2886^***^	0.3592^***^	1							
7. DEBT	0.4051	0.2076	0.0282	0.3933	1.4486	0.0176^**^	−0.0643^***^	−0.3270^***^	−0.1193^***^	0.4870^***^	0.3486^***^	1						
8. FA	0.2200	0.1526	0.0021	0.1902	0.6984	−0.0513^***^	0.0062	−0.0982^***^	−0.1860^***^	0.1358^***^	0.2001^***^	0.1737^***^	1					
9. IA	0.0328	0.0755	0	0.0010	0.5060	0.0442^***^	−0.0339^***^	−0.0559^***^	0.2305^***^	0.0160^**^	−0.2168^***^	−0.1278^***^	−0.2404^***^	1				
10. PR	0.1706	0.1258	0.0153	0.1368	0.7907	0.0269^***^	0.0342^***^	−0.0245^***^	−0.0171^**^	−0.3093^***^	−0.1847^***^	−0.2977^***^	−0.1848^***^	0.1616^***^	1			
11. NPR	2.7687	3.2177	0.2001	1.7426	34.6088	−0.0413^***^	0.0223^***^	0.1832	0.0226^***^	−0.3330^***^	−0.2199^***^	−0.6199^***^	−0.2681^***^	−0.0572^***^	0.2371^***^	1		
12. BOARD	2.3708	0.2199	1.7917	2.3025	2.9957	0.0275^***^	0.0011	−0.0617^***^	−0.0984^***^	0.2512^***^	0.2502^***^	0.1719^***^	0.1341^***^	−0.0340^***^	−0.0649^***^	−0.1335^***^	1	
13. TOP1	34.7250	14.5272	8.3200	32.9800	76.9500	−0.0137^**^	0.0262^***^	0.1267^***^	−0.0389^***^	0.1791^***^	0.1835^***^	0.0422^***^	0.0818^***^	−0.1803^***^	−0.1239^***^	−0.1239^***^	−0.0173^**^	1

^*^, ^**^, and ^***^ indicate significant correlation at the level of 10%, 5%, and 1%, respectively.

### Analysis of regression results

Table 3 lists the test results of the relationship between multiple performance feedback and EI. Model 2 indicates that the multiple performance feedback in the cases of consistency has a significant positive impact on the EI of enterprises (*a*_1_ = 0.1196, *p* < 0.01), indicating that in the cases of consistency, the historical performance shortfall-industry performance shortfall is more conducive to the enterprises’ EI, H1 is supported. Model 4 indicates that multiple performance feedback in the cases of inconsistency has a significant positive impact on the EI of enterprises (*a*_2_ = 0.2516, *p* < 0.01), indicating that in the cases of inconsistency, the historical performance shortfall-industry performance surplus is more conducive to the enterprises’ EI, supporting H2.

**Table 3:** Multiple performance feedback and enterprises’ exploratory innovation.

Variables	(M1)	(M2)	(M3)	(M4)
	EI	EI	EI	EI
HAPIAP1	0.2022^***^ (0.018)	0.1196^***^ (0.018)		
HAPIAP2			0.6309^***^ (0.031)	0.2516^***^ (0.033)
SCALE		0.5478^***^ (0.013)		0.7185^***^ (0.022)
STATE		−0.0218 (0.059)		−0.1830^**^ (0.091)
DEBT		−0.0776 (0.072)		−0.4594^***^ (0.122)
FA		0.1362 (0.096)		0.5813^***^ (0.147)
IA		−1.0906^***^ (0.140)		−1.2624^***^ (0.215)
PR		0.0002 (0.000)		0.0006^*^ (0.000)
NPR		−0.0231^***^ (0.004)		−0.0304^***^ (0.004)
BOARD		0.1566^***^ (0.042)		0.1185^*^ (0.062)
TOP1		−0.0143^***^ (0.001)		−0.0130^***^ (0.002)
Constant	2.1865^***^ (0.013)	−9.7004^***^ (0.309)	2.1841^***^ (0.012)	−13.0793^***^ (0.495)
ID	YES	YES	YES	YES
YEAR	YES	YES	YES	YES
Observations	8,815	8,815	6,010	6,010
*R*-squared	0.012	0.251	0.074	0.366

^*^, ^**^, and ^***^ indicate significant correlation at the level of 10%, 5%, and 1%, respectively.

The test results of the moderating effect of RI are shown in Table 4. Model 2 analyzes the moderating effect of RI on the relationship between the multiple performance feedback in the cases of consistency and enterprises’ EI. The empirical analysis results of Model 2 show that it has a significant positive moderating effect (*b*_13_ = 0.0440, *p* < 0.01), indicating that in the cases of consistency, the RI positively enhances the relationship between the historical performance shortfall-industry performance shortfall and the enterprises’ EI, H3 is supported. Model 4 analyzes the moderating effect of RI on the relationship between the multiple performance feedback in the cases of inconsistency and enterprises’ EI. The empirical analysis results of Model 4 show that it has a significant positive moderating effect (*b*_23_ = 0.0669, *p* < 0.01), indicating that in the cases of inconsistency, RI positively enhances the relationship between the historical performance shortfall-industry performance surplus and enterprises’ EI, supporting H4.

**Table 4:** Multiple performance feedback, regional institutional development, and enterprises’ exploratory innovation.

Variables	(M1)	(M2)	(M3)	(M4)
	EI	EI	EI	EI
HAPIAP1	0.0896^***^ (0.015)	0.0771^***^ (0.017)		

HAPIAP1*RI	0.0356^***^ (0.007)	0.0440^***^ (0.008)		

HAPIAP2			0.1342^***^ (0.027)	0.1091^***^ (0.032)
HAPIAP2*RI			0.0998^***^ (0.012)	0.0669^***^ (0.014)
RI	0.4889^***^ (0.008)	0.3870^***^ (0.012)	0.5546^***^ (0.011)	0.3547^***^ (0.018)
SCALE		0.2341^***^ (0.016)		0.3763^***^ (0.027)
STATE		0.0564 (0.055)		−0.0979 (0.086)
DEBT		0.0230 (0.067)		−0.2536^**^ (0.116)
FA		0.1902^**^ (0.090)		0.3042^**^ (0.141)
IA		−0.8909^***^ (0.131)		−1.0524^***^ (0.205)
PR		0.0002 (0.000)		0.0005 (0.000)
NPR		−0.0096^***^ (0.004)		−0.0203^***^ (0.004)
BOARD		0.1136^***^ (0.039)		0.0740 (0.059)
TOP1		−0.0060^***^ (0.001)		−0.0081^***^ (0.002)
Constant	2.2703^***^ (0.011)	−2.9893^***^ (0.353)	2.3065^***^ (0.011)	−5.6244^***^ (0.602)
ID	YES	YES	YES	YES
YEAR	YES	YES	YES	YES
Observations	8,815	8,815	6,010	6,010
*R*-squared	0.323	0.342	0.377	0.424

^*^, ^**^, and ^***^ indicate significant correlation at the level of 10%, 5% and 1%, respectively.

### Robustness tests

We also conducted the following tests to ensure the robustness of the research conclusions. First, we replaced the measurement indicator of the independent variable. We used ROA to measure the performance of the enterprise. To ensure the robustness of the research conclusions, we change the performance measurement indicator to further test the hypothesis. We replace the enterprise performance measurement indicator with OROA, namely, the operating profit margin on total assets. Table 5 shows the regression results of the robustness tests. Model 2 indicates that the multiple performance feedback in the cases of consistency has a significant positive impact on enterprises’ EI (*a*_1_ = 1.8230, *p* < 0.01), indicating that a positive relationship exists between the historical performance shortfall-industry performance shortfall and enterprises’ EI. Model 4 indicates that the multiple performance feedback in the cases of inconsistency has a significant positive impact on enterprises’ EI (*a*_2_ = 0.1559, *p* < 0.01), indicating a positive relationship between the historical performance shortfall-industry performance surplus and enterprises’ EI, which was similar to our assumptions. This indicates that our research conclusions have high robustness.

**Table 5:** Robustness test: replacing the measurement indicator of performance feedback.

Variables	(M1) OROA	(M2) OROA	(M3) OROA	(M4) OROA
	EI	EI	EI	EI
HAPIAP1	3.1069^***^ (0.507)	1.8230^***^ (0.551)		
HAPIAP2			0.4700^***^ (0.028)	0.1559^***^ (0.029)
SCALE		0.6418^***^ (0.016)		0.7230^***^ (0.022)
STATE		−0.0250 (0.069)		−0.1552^*^ (0.091)
DEBT		−0.2486^***^ (0.086)		−0.3872^***^ (0.121)
FA		0.2119^**^ (0.108)		0.6792^***^ (0.146)
IA		−1.2430^***^ (0.179)		−1.1970^***^ (0.215)
PR		0.0002 (0.000)		0.0006* (0.000)
NPR		−0.0217^***^ (0.004)		−0.0304^***^ (0.004)
BOARD		0.1497^***^ (0.046)		0.1255^**^ (0.062)
TOP1		−0.0160^***^ (0.001)		−0.0138^***^ (0.002)
Constant	2.3152^***^ (0.007)	−11.5481^***^ (0.366)	2.2644^***^ (0.011)	−13.1807^***^ (0.497)
ID	YES	YES	YES	YES
YEAR	YES	YES	YES	YES
Observations	8,815	8,815	6,010	6,010
*R*-squared	0.004	0.257	0.049	0.361

^*^, ^**^, and ^***^ indicate significant correlation at the level of 10%, 5%, and 1%, respectively.

Second, we modified the measurement method of the independent variable. When we measured the performance feedback, the actual performance weight in the calculation formula of the aspiration level was set to 0.6. Considering that different weight settings will affect the results of the aspiration level, we set the actual performance weight in the aspiration level to 0.5. The results were still significant. Table 6 shows the regression results of robustness tests. Model 2 indicates that the multiple performance feedback in the cases of consistency has a significant positive impact on enterprises’ EI (*a*_1_ = 0.1345, *p* < 0.01), indicating that the historical performance shortfall-industry performance shortfall is more conducive to the enterprises’ EI. Model 4 shows that the multiple performance feedback in the cases of inconsistency has a significant positive impact on enterprises’ EI (*a*_2_ = 0.2171, *p* < 0.01), indicating that the historical performance shortfall-industry performance surplus is more conducive to the enterprises’ EI.

**Table 6:** Robustness test: replacing the measurement method of performance feedback.

Variables	(M1) 0.5	(M2) 0.5	(M3) 0.5	(M4) 0.5
	EI	EI	EI	EI
HAPIAP1	0.2516^***^ (0.017)	0.1345^***^ (0.017)		
HAPIAP2			0.5823^***^ (0.030)	0.2171^***^ (0.031)
SCALE		0.5410^***^ (0.013)		0.7160^***^ (0.022)
STATE		−0.0205 (0.059)		−0.1635^*^ (0.091)
DEBT		−0.0721 (0.071)		−0.4137^***^ (0.121)
FA		0.1301 (0.096)		0.6319^***^ (0.146)
IA		−1.0752^***^ (0.140)		−1.2369^***^ (0.215)
PR		0.0002 (0.000)		0.0006^*^ (0.000)
NPR		−0.0230^***^ (0.004)		−0.0305^***^ (0.004)
BOARD		0.1523^***^ (0.042)		0.1193^*^(0.062)
TOP1		−0.0141^***^ (0.001)		−0.0136^***^ (0.002)
Constant	2.1680^***^ (0.012)	−9.5510^***^ (0.310)	2.2160^***^ (0.012)	−13.0222^***^ (0.497)
ID	YES	YES	YES	YES
YEAR	YES	YES	YES	YES
Observations	8,815	8,815	6,010	6,010
*R*-squared	0.021	0.253	0.067	0.364

^*^, ^**^, and ^***^ indicate significant correlation at the level of 10%, 5%, and 1%, respectively.

Finally, considering the problem of endogeneity, the performance feedback of the enterprise affects the EI of the enterprise. In turn, the EI of the enterprise affects the business performance, and subsequently affects the performance feedback. To reduce the impact caused by endogeneity, we constructed a dynamic panel model with a lag of one period of enterprise EI and used the systematic GMM estimation method, which can control endogeneity (Wan et al., 2021). Table 7 shows the regressions results of the robustness tests. Model 1 indicates that the multiple performance feedback in the cases of consistency has a significant positive impact on enterprises’ EI (*a*_1_ = 0.2163, *p* < 0.05), and Model 2 shows that the multiple performance feedback in the cases of inconsistency has a significant positive impact on enterprises’ EI (*a*_2_ = 0.2609, *p* < 0.05), which is the same as our assumptions, indicating that our research conclusions are still highly robust after controlling endogeneity to a certain extent.

**Table 7:** Robustness test: GMM model.

Variables	(M1)	(M2)
	EI	EI
L.EI	1.0189^***^ (0.005)	1.0002^***^ (0.014)
HAPIAP1	0.2163^**^ (0.102)	
HAPIAP2		0.2609^**^ (0.125)
Control	YES	YES
ID	YES	YES
YEAR	YES	YES

^*^, ^**^, and ^***^ indicate significant correlation at the level of 10%, 5%, and 1%, respectively.

## Conclusion and discussion

### Conclusion

We used the data of Chinese A-share listed companies from 2008 to 2019 as samples for hypothesis testing and drew the following conclusions: (1) In the cases of consistent performance feedback, compared with the scenario of historical performance surplus-industry performance surplus, historical performance shortfall-industry performance shortfall is more likely to promote EI, which is conducive to sustainable growth. In the scenario of historical performance shortfall-industry performance shortfall, enterprises need to adjust innovation strategy to adapt to the competition of customers and the product market. To actively change the shortfall state of enterprises and obtain excess profits, decision-makers are more willing to try risk-taking or innovative behaviors, thus promoting the EI of enterprises. (2) In the cases of inconsistent performance feedback, compared with the scenario of historical performance surplus-industry performance shortfall, historical performance shortfall-industry performance surplus is more likely to promote EI. In the scenario of historical performance shortfall-industry performance surplus, enterprises may lack sufficient resource input, resulting in an unsustainable competitive advantage. To narrow the historical performance shortfall and secure the position of the industry, enterprises will actively conduct EI to provide the impetus for the healthy development of enterprises. (3) In the cases of consistent performance feedback, especially in the scenario of historical performance shortfall-industry performance shortfall, decision-makers of enterprises make full use of the RI to obtain more policy support and market information, which can effectively reduce the cost of enterprise problematic search and improve the enthusiasm of enterprises’ EI. RI strengthens the positive impact of the historical and industry performance shortfall on enterprises’ EI. (4) In the cases of inconsistent performance feedback, especially in the scenario of historical performance shortfall-industry performance surplus, the optimized RI weakens decision-makers’ perception of external environment uncertainty and improves enterprises’ confidence in EI. RI enhances the positive impact of the historical performance shortfall-industry performance surplus on enterprises’ EI.

### Management implications

Our study has the following implications for management: (1) Enterprises usually revise strategic decisions based on the experience of learning as an adaptive rational system. The performance feedback mechanism provides an important reference point for enterprises. In the actual operation of enterprises, decision-makers should adopt a positive attitude when selecting the reference points, and rationally change the strategic decision of enterprises according to the performance feedback. Concerning the reference to the multiple enterprise expectation gap, enterprises should constantly optimize resource allocation and actively obtain external information, knowledge, and other resources to achieve the target aspiration level. (2) Decision-makers must treat the inconsistent performance feedback rationally. When faced with fuzzy performance feedback signals, decision-makers should reduce the self-enhancing effect. Considering self-reputation, managers attach great importance to historical performance feedback. Industry performance feedback can reflect the industry’s competitive position, which plays an essential role in the long-term development of enterprises. Decision-makers should pay attention to the impact of historical performance feedback on enterprises and improve the importance of industry performance feedback. Enterprise should make full use of the feedback results of consistency and inconsistency to promote EI and enhance the ability of enterprises to adapt to the external environment, achieving sustainable growth of enterprises. (3) In the scenario of industry performance surplus-historical performance feedback surplus, the enterprise is in a state of absolute benefit. In this scenario, decision-makers should make full use of the redundant resource search motivation, improve the EI ability of enterprises, and change the tendency of invariability when enterprises are flourishing. In the scenario of industry performance shortfall-historical performance shortfall, the enterprise is in a state of complete loss. In this scenario, decision-makers should take advantage of the problem to search for motivation and actively seek breakthroughs. Enterprises should further optimize the allocation of resources, acquire external knowledge and technology, and enhance their EI ability. (4) When making innovation decisions, enterprises should not only consider the impact of performance feedback on EI, but also pay attention to external regional institutional development, actively use the convenience brought by RI for EI, and reduce the cost of EI and promote the sustainable development of the enterprise.

### Limitations and future research

Our study has the following limitations. We divided the cases of inconsistency based on the direction of surplus or shortfall, exploring the impact of different cases of historical performance feedback and industry performance feedback on enterprises’ EI. However, performance feedback is not only inconsistent in terms of direction, but may also inconsistent in intensity. For example, in the case of surplus or shortfall, there are differences in the intensity of historical and industry performance surplus, which should be discussed in future research. When selecting the regulatory variable, we perform an in-depth analysis of the RI of the relationship between performance feedback and EI, from the perspective of transformation in China. However, we did not fully consider the contingency influence of internal factors, such as precipitated redundant resources or non-precipitated redundant resources, this issue can be taken up by future research.

## Data availability statement

The data supporting the conclusions of this article will be made available by the authors, without undue reservation.

## Author contributions

XS collected literature. WF designed the research and wrote the manuscript. XS and WF performed the empirical analysis. All authors contributed to the article and approved the submitted version.

## Funding

This research was supported by the National Social Science Foundation of China (19BGL150).

## Conflict of interest

The authors declare that the research was conducted in the absence of any commercial or financial relationships that could be construed as a potential conflict of interest.

## Publisher’s note

All claims expressed in this article are solely those of the authors and do not necessarily represent those of their affiliated organizations, or those of the publisher, the editors and the reviewers. Any product that may be evaluated in this article, or claim that may be made by its manufacturer, is not guaranteed or endorsed by the publisher.

## References

[ref1] Aghion P. Bloom N. Blundell R. Griffith R. Howitt P. (2005). Competition and innovation: an inverted u relationship. Q. J. Econ. 120, 701–728. doi: 10.1093/qje/120.2.701

[ref2] Alam A. Uddin M. Yazdifar H. (2019). Institutional determinants of R&D investment: evidence from emerging markets. Technol. Forecast. Soc. Change 138, 34–44. doi: 10.1016/j.techfore.2018.08.007

[ref3] Arzubiaga U. Maseda A. Iturralde T. (2019). Exploratory and exploitative innovation in family businesses: the moderating role of the family firm image and family involvement in top management. Rev. Manage. Sci. 13, 1–31. doi: 10.1007/s11846-017-0239-y1989

[ref4] Audia P. G. Brion S. Greve H. R. (2015). Self-assessment, self-enhancement, and the choice of comparison organizations for evaluating organizational performance. Adv. Strateg. Manage. 32, 89–118. doi: 10.1108/S0742-332220150000032018

[ref5] Barnett W. P. Pontikes E. G. (2008). The red queen, success bias, and organizational inertia. Manage. Sci. 54, 1237–1251. doi: 10.1287/mnsc.1070.0808

[ref6] Ben-Oz C. Greve H. R. (2015). Short-and long-term performance feedback and absorptive capacity. J. Manage. 41, 1827–1853. doi: 10.1177/0149206312466148

[ref7] Berraies S. (2019). The effect of enterprise social networks use on exploitative and exploratory innovations: mediating effect of sub-dimensions of intellectual capital. J. Intellect. Cap. 20, 426–452. doi: 10.1108/JIC-02-2019-0030

[ref8] Blagoeva R. R. Mom T. J. M. Jansen J. J. P. George G. (2020). Problem-solving or self-enhancement? A power perspective on how CEOs affect R&D search in the face of inconsistent feedback. Acad. Manage. J. 63, 332–355. doi: 10.5465/amj.2017.0999

[ref9] Bonaime A. Gulen H. Ion M. (2018). Does policy uncertainty affect mergers and acquisitions? J. Financ. Econ. 129, 531–558. doi: 10.1016/j.jfineco.2018.05.007

[ref10] Chen W.-R. (2008). Determinants of firms’ backward-and forward-looking R&D search behavior. Organ. Sci. 19, 609–622. doi: 10.1287/orsc.1070.0320

[ref11] Chen J. Yin X. Fu X. Bruce M. K. (2021). Beyond catch-up: could China become the global innovation powerhouse? China’s innovation progress and challenges from a holistic innovation perspective. Ind. Corp. Chang. 30, 1037–1064. doi: 10.1093/icc/dtab032

[ref12] Chen W. Zhong X. Lv D. D. (2022). Negative performance feedback, CEO tenure, and punctuated equilibrium innovation. R D Manage. 52, 564–576. doi: 10.1111/radm.12501

[ref13] Chng D. H. M. Shih E. Rodgers M. S. Song X.-B. (2015). Managers’ marketing strategy decision making during performance decline and the moderating influence of incentive pay. J. Acad. Mark. Sci. 43, 629–647. doi: 10.1007/s11747-014-0401-x

[ref14] Choi J. Rhee M. Kim Y.-C. (2019). Performance feedback and problemistic search: the moderating effects of managerial and board outsiderness. J. Bus. Res. 102, 21–33. doi: 10.1016/j.jbusres.2019.04.039

[ref15] Chu C.-C. Li Y.-L. Li S.-J. Ji Y. (2021). Uncertainty, venture capital and entrepreneurial enterprise innovation-evidence from companies listed on China’s GEM. Pacific Basin Financ. J. 68:101576. doi: 10.1016/j.pacfin.2021.101576

[ref16] Chung D. Shin D. (2021). When do firms invest in R&D? Two types of performance feedback and organizational search in the Korean shipbuilding industry. Asian Bus. Manage. 20, 583–617. doi: 10.1057/s41291-019-00102-1

[ref17] Ciftci M. Cready W. M. (2011). Scale effects of R&D as reflected in earnings and returns. J. Account. Econ. 52, 62–80. doi: 10.1016/j.jacceco.2011.02.003

[ref18] Cyert R. M. March J. G. (1963). A Behavioral Theory of the Firm. Englewood Cliffs: NJ: Prentice-Hall.

[ref19] Davis L. North D. (1970). Institutional change and American economic growth: a first step towards a theory of institutional innovation. J. Econ. Hist. 30, 131–149. doi: 10.1017/S0022050700078633

[ref20] Denrell J. March J. G. (2001). Adaptation as information restriction: the hot stove effect. Organ. Sci. 12, 523–538. doi: 10.1287/orsc.12.5.523.10092

[ref21] Desai V. (2014). Learning to behave badly: performance feedback and illegal organizational action. Ind. Corp. Chang. 23, 1327–1355. doi: 10.1093/icc/dtt043

[ref22] Donbesuur F. Ampong G. O. A. Owusu-Yirenkyi D. Chu I. (2020). Technological innovation, organizational innovation and international performance of SMEs: the moderating role of domestic institutional environment. Technol. Forecast. Soc. Change 161:120252. doi: 10.1016/j.techfore.2020.120252

[ref23] Eggers J. P. Suh J.-H. (2019). Experience and behavior: how negative feedback in new versus experienced domains affects firm action and subsequent performance. Acad. Manage. J. 62, 309–334. doi: 10.5465/amj.2017.0046

[ref24] Gaba V. Joseph J. (2013). Corporate structure and performance feedback: aspirations and adaptation in M-form firms. Organ. Sci. 24, 1102–1119. doi: 10.1287/orsc.1120.0788

[ref25] Gao Y. Gao S. Zhou Y. Huang K.-F. (2015). Picturing firms’ institutional capital-based radical innovation under China’s institutional voids. J. Bus. Res. 68, 1166–1175. doi: 10.1016/j.jbusres.2014.11.011

[ref26] Gao Y. Hu Y. Liu X. Zhang H. (2021). Can public R&D subsidy facilitate firms’ exploratory innovation? The heterogeneous effects between central and local subsidy programs. Res. Policy 50:104221. doi: 10.1016/j.respol.2021.104221

[ref27] Gilsing V. Nooteboom B. Vanhaverbeke W. Duysters G. van den Oord A. (2008). Network embeddedness and the exploration of novel technologies: technological distance, betweenness centrality and density. Res. Policy 37, 1717–1731. doi: 10.1016/j.respol.2008.08.010

[ref28] Greve H. (2003). Investment and the behavioral theory of the firm: evidence from shipbuilding. Ind. Corp. Chang. 12, 1051–1076. doi: 10.1093/icc/12.5.1051

[ref29] Guan J. Liu N. (2016). Exploitative and exploratory innovations in knowledge network and collaboration network: a patent analysis in the technological field of nano-energy. Res. Policy 45, 97–112. doi: 10.1016/j.respol.2015.08.002

[ref30] Hart O. Moore J. (2008). Contracts as reference points. Q. J. Econ. 123, 1–48. doi: 10.1162/qjec.2008.123.1.1

[ref31] He L. Huang L. Yang G. (2021). Invest in innovation or not? How managerial cognition and attention allocation shape corporate responses to performance shortfalls. Manage. Organ. Rev. 17, 815–850. doi: 10.1017/mor.2021.58

[ref32] Honig B. Samuelsson M. (2021). Business planning by intrapreneurs and entrepreneurs under environmental uncertainty and institutional pressure. Technovation 99:102124. doi: 10.1016/j.technovation.2020.102124

[ref33] Jordan A. H. Audia P. G. (2012). Self-enhancement and learning from performance feedback. Acad. Manage. 37, 211–231. doi: 10.5465/amr.2010.0108

[ref34] Joseph J. Gaba V. (2015). The fog of feedback: ambiguity and firm responses to multiple aspiration levels. Strateg. Manage. J. 36, 1960–1978. doi: 10.1002/smj.2333

[ref35] Joseph J. Klingebiel R. Wilson A. J. (2016). Organizational structure and performance feedback: centralization, aspirations, and termination decisions. Organ. Sci. 27, 1065–1083. doi: 10.1287/orsc.2016.1076

[ref36] Kacperczyk A. Beckman C. M. Moliterno T. P. (2015). Disentangling risk and change: internal and external social comparison in the mutual fund industry. Admin. Sci. Q. 60, 228–262. doi: 10.1177/0001839214566297

[ref37] Kim J.-Y. J. Finkelstein S. Haleblian J. J. (2015). All aspirations are not created equal: the differential effects of historical and social aspirations on acquisition behavior. Acad. Manage. J. 58, 1361–1388. doi: 10.5465/amj.2012.1102

[ref38] Lin W.-T. (2014). How do managers decide on internationalization processes? The role of organizational slack and performance feedback. J. World Bus. 49, 396–408. doi: 10.1016/j.jwb.2013.08.001

[ref39] Lin M. Patel P. C. (2019). Distant search, technological diversity, and branding focus: incremental and radical innovation in small-and medium-sized consignees. IEEE Trans. Eng. Manage. 66, 170–179. doi: 10.1109/TEM.2018.2836179

[ref40] Liu H. Jiang J. Xue R. Meng X. Hu S. (2022). Corporate environmental governance scheme and investment efficiency over the course of COVID-19. Financ. Res. Lett. 47:102726. doi: 10.1016/j.frl.2022.102726, PMID: 35185400 PMC8842463

[ref41] Liu H. Wang Y. Xue R. Linnenluecke M. Cai C. W. (2021). Green commitment and stock prick crash risk. Financ. Res. Lett. 47:102646. doi: 10.1016/j.frl.2021.102646

[ref42] Lu L.-H. Wong P.-K. (2019). Performance feedback, financial slack and the innovation behavior of firms. Asia Pacific J. Manage. 36, 1079–1109. doi: 10.1007/s10490-018-9634-4

[ref43] Lucas G. J. M. Knoben J. Meeus M. T. H. (2015). Contradictory yet coherent? Inconsistency in performance feedback and R&D investment change. J. Manage. 44, 658–681. doi: 10.1177/0149206315584821

[ref44] Lv D. D. Chen W. Zhu H. Lan H. (2019). How does inconsistent negative performance feedback affect the R&D investments of firms? A study of publicly listed firms. J. Bus. Res. 102, 151–162. doi: 10.1016/j.jbusres.2019.04.045

[ref45] O’Brien J. P. David P. (2014). Reciprocity and R&D search: applying the behavioral theory of the firm to a communitarian context. Strateg. Manage. J. 35, 550–565. doi: 10.1002/smj.2105

[ref46] Oliver C. (1992). The antecedents of deinstitutionalization. Organ. Stud. 13, 563–588. doi: 10.1177/017084069201300403

[ref47] Parker O. N. Krause R. Covin J. G. (2017). Ready, set, slow: how aspiration-relative product quality impacts the rate of new product introduction. J. Manage. 43, 2333–2356. doi: 10.1177/0149206315569314

[ref48] Saraf N. Dasgupta S. Blettner D. P. (2021). How do managerial perceptions of performance feedback affect innovation? Strateg. Organ. 20, 451–480. doi: 10.1177/14761270211019484

[ref49] Shi Q. Shan Y. Zhong C. Cao Y. Xue R. (2022). How would GVCs participation affect carbon intensity in the? “Belt and road initiative” countries? Energy Econ. 111:106075. doi: 10.1016/j.eneco.2022.106075

[ref50] Shu C. Zhou K. Z. Xiao Y. Gao S. (2016). How green management influences product innovation in China: the role of institutional benefits. J. Bus. Ethics 133, 471–485. doi: 10.1007/s10551-014-2401-7

[ref51] Slavova K. Jong S. (2021). University alliances and firm exploratory innovation: evidence from therapeutic product development. Technovation 107:102310. doi: 10.1016/j.technovation.2021.102310

[ref52] Su Y. Si S. (2015). What motivates financial innovation across countries? The influences of performance aspiration and economic freedom. Manage. Int. Rev. 55, 563–587. doi: 10.1007/s11575-014-0237-0

[ref53] Su X. Zhou S. Xue R. Tian J. (2020). Does economic policy uncertainty raise corporate precautionary cash holdings? Evidence from China. Account. Financ. 60, 4567–4592. doi: 10.1111/acfi.12674

[ref54] Szczygielski K. Grabowski W. Pamukcu M. T. Tandogan V. S. (2017). Does government support for private innovation matter? Firm-level evidence from two catching-up countries. Res. Policy 46, 219–237. doi: 10.1016/j.respol.2016.10.009

[ref55] Tang Y. Hu X. Petti C. Thürer M. (2019). Institutional incentives and pressures in Chinese manufacturing firms’ innovation. Manage. Decis. 58, 812–827. doi: 10.1108/MD-08-2018-0933

[ref56] Tian J.-F. Pan C. Xue R. Yang X.-T. Wang C. Ji X.-Z.. (2020). Corporate innovation and environmental investment: the moderating role of institutional environment. Adv. Clim. Chang. Res. 11, 85–91. doi: 10.1016/j.accre.2020.05.003

[ref57] Tian X. Wang T. Y. (2014). Tolerance for failure and corporate innovation. Rev. Financ. Stud. 27, 211–255. doi: 10.1093/rfs/hhr130

[ref58] Tian J. Yu L. Xue R. Zhuang S. Shan Y. (2022). Global low-carbon energy transition in the post-COVID-19 era. Appl. Energy 307:118205. doi: 10.1016/j.apenergy.2021.118205, PMID: 34840400 PMC8610812

[ref59] Wan L. Li R. Chen Y. (2022). Negative performance feedback and corporate venture capital: the moderating effect of CEO overconfidence. Appl. Econ. 54, 1829–1843. doi: 10.1080/00036846.2021.1982133

[ref60] Wan D. Xue R. Linnenluecke M. Tian J. Shan Y. (2021). The impact of investor attention during COVID-19 on investment in clean energy versus fossil fuel firms. Financ. Res. Lett. 43:101955. doi: 10.1016/j.frl.2021.101955 PMC9665964 36406287

[ref61] Wang X. Lou T. (2020). The effect of performance feedback on firms’ unplanned marketing investments. J. Bus. Res. 118, 441–451. doi: 10.1016/j.jbusres.2020.07.015

[ref62] Wang J. J. Shi W. Lin Y. Yang X. (2020). Relational ties, innovation, and performance: a tale of two pathways. Ind. Mark. Manage. 89, 28–39. doi: 10.1016/j.indmarman.2020.06.007

[ref63] Wang D. Su Z. Guo H. (2019). Top management team conflict and exploratory innovation: the mediating impact of market orientation. Ind. Mark. Manage. 82, 87–95. doi: 10.1016/j.indmarman.2019.02.014

[ref64] Wang X. Wang L. (2020). Enterprise performance gap and green innovation: a contingency thought on decision convention of “poor performance leads to change”. J. Shanghai Univ. Financ. Econ. 22, 18–33. doi: 10.16538/j.cnki.jsufe.2020.01.002

[ref65] Wang Y. Wang X. Xu W. (2021). How does negative performance feedback affect a firm’s openness in its innovation search behaviour? The moderating role of organisational slack. Technol. Anal. Strateg. Manage., 1–14. doi: 10.1080/09537325.2021.1976405 [Epub ahead of print]

[ref66] Wang N. Xiao M. Savin I. (2021). Complementarity effect in the innovation strategy: internal R&D and acquisition of capital with embodied technology. J. Technol. Transf. 46, 459–482. doi: 10.1007/s10961-020-09780-y

[ref67] Wang C. Yi J. Kafouros M. Yan Y. (2015). Under what institutional conditions do business groups enhance innovation performance? J. Bus. Res. 68, 694–702. doi: 10.1016/j.jbusres.2014.08.002

[ref68] Wu J. Ma Z. Liu Z. Lei C. K. (2019). A contingent view of institutional environment, firm capability, and innovation performance of emerging multinational enterprises. Ind. Mark. Manage. 82, 148–157. doi: 10.1016/j.indmarman.2019.01.018

[ref69] Xiao Y. Liu S. Dai T. (2021). Positive and negative supervisor development feedback, team harmonious innovation passion and team creativity. Front. Psychol. 12:681910. doi: 10.3389/fpsyg.2021.681910, PMID: 34539486 PMC8444987

[ref70] Xu K. Huang K.-F. Gao S. (2012). The effect of institutional ties on knowledge acquisition in uncertain environments. Asia Pacific J. Manage. 29, 387–408. doi: 10.1007/s10490-010-9196-6

[ref71] Xu C. Zhang H. Wang M. Iqbal A. (2022). Investigating the relationship between entity financialization, managers’ incentives, and enterprise’s innovation: fresh evidence from China. Front. Psychol. 12:810294. doi: 10.3389/fpsyg.2021.810294, PMID: 35308072 PMC8931463

[ref72] Xue R. Qian G. Qian Z. Li L. (2021). Entrepreneurs’ implicit and explicit achievement motives and their early international commitment. Manage. Int. Rev. 61, 91–121. doi: 10.1007/s11575-020-00436-5

[ref73] Xue R. Qian G. Qian Z. Li L. (2022). How do foreign customers’ perceptions of product-harm crises affect their transfer of capability- and character-based stigma? Int. Mark. Rev. 39, 120–141. doi: 10.1108/IMR-09-2020-0197

[ref74] Yang C.-H. Tseng Y.-H. Chen C.-P. (2012). Environmental regulations, induced R&D, and productivity: evidence from Taiwan’s manufacturing industries. Resour. Energy Econ. 34, 514–532. doi: 10.1016/j.reseneeco.2012.05.001

[ref75] Yang Z. Zhang H. Xie E. (2017). Performance feedback and supplier selection: a perspective from the behavioral theory of the firm. Ind. Mark. Manage. 63, 105–115. doi: 10.1016/j.indmarman.2016.12.003

[ref76] Ye H. Zhao Y. (2021). An emprirical study on the impact of inconsistent multiple performance feedback on enterprise’s technological innovation. Technol. Innov. Manage. 42, 368–375. doi: 10.14090/j.cnki.jscx.2021.0402

[ref77] Yi Y. Ji J. Lyu C. (2022). How do polychronicity and interfunctional coordination affect the relationship between exploratory innovation and the quality of new product development? J. Knowl. Manage. 26, 1687–1704. doi: 10.1108/JKM-04-2021-0292

[ref78] Yu C. Wang T. Gu X. (2022). Collective reputation cognition, network competence and enterprise innovation performance. Manage. Decis. 60, 567–588. doi: 10.1108/MD-10-2019-1420

[ref79] Zajac E. J. Kraatz M. S. (1993). A diametric forces model of strategic change: assessing the antecedents and consequences of restructuring in the higher education industry. Strateg. Manage. J. 14, 83–102. doi: 10.1002/smj.4250140908

[ref80] Zhang Y. Gong Y. (2018). Stock return or sales growth? Multiple performance feedback and strategic investments under securities analysts’ earnings pressure. J. Manage. Stud. 55, 1356–1385. doi: 10.1111/joms.12392

[ref81] Zhang Y. Zhuo C. Deng F. (2022). Policy uncertainty, financialization and enterprise technological innovation: a way forward towards economic development. Front. Environ. Sci. 10:905505. doi: 10.3389/fenvs.2022.905505

[ref82] Zhong X. Chen W. Ren G. (2022). The effects of performance shortfalls on firms’ exploitation and exploration R&D internationalization decisions: does industry environmental matter? Technovation 112:102408. doi: 10.1016/j.technovation.2021.102408

[ref83] Zhong X. Ren L. Song T. (2021). Beyond market strategies: how multiple decision-maker groups jointly influence underperforming firms’ corporate social (Ir)responsibility. J. Bus. Ethics 178, 481–499. doi: 10.1007/s10551-021-04796-2

